# The relationship between the gut microbiome and mild cognitive impairment in patients without dementia: a cross-sectional study conducted in Japan

**DOI:** 10.1038/s41598-019-55851-y

**Published:** 2019-12-18

**Authors:** Naoki Saji, Kenta Murotani, Takayoshi Hisada, Tsuyoshi Tsuduki, Taiki Sugimoto, Ai Kimura, Shumpei Niida, Kenji Toba, Takashi Sakurai

**Affiliations:** 1 Center for Comprehensive Care and Research on Memory Disorders, National Center for Geriatrics and Gerontology, Aichi, Japan; 2 Biostatistics Center, Graduate School of Medicine, Kurume University, Fukuoka, Japan; 3 TechnoSuruga Laboratory Co., Ltd, Shizuoka, Japan; 4 Laboratory of Food and Biomolecular Science, Department of Bioscience and Biotechnology for Future Bioindustries, Graduate School of Agricultural Science, Tohoku University, Miyagi, Japan; 5 Medical Genome Center, National Center for Geriatrics and Gerontology, Aichi, Japan; 6 Department of Cognition and Behavioural Science, Nagoya University Graduate School of Medicine, Aichi, Japan

**Keywords:** Microbiology, Dementia, Risk factors

## Abstract

Recent studies have revealed an association between the dysregulation of the gut microbiome and dementia. However, whether this dysregulation is associated with mild cognitive impairment (MCI), an early stage of cognitive decline, in patients without dementia remains unclear. We performed a cross-sectional analysis to determine the association between the gut microbiome and MCI. Data, including patient demographics, risk factors, cognitive function, and brain imaging, were collected. The gut microbiome was assessed through terminal restriction fragment length polymorphism analysis. Multivariable logistic regression models were used to identify factors independently associated with MCI. Graphical modelling was used to illustrate mutual associations between MCI and identified factors. We analysed 82 patients, 61 of whom exhibited MCI. Patients with MCI had a higher prevalence of *Bacteroides*. Furthermore, patients with more *Bacteroides* were more likely to present with white matter hyperintensity and high voxel-based specific regional analysis system for Alzheimer’s Disease (VSRAD) scores, indicating cortical and hippocampal atrophy. A multivariable logistic regression analysis revealed that a greater prevalence of *Bacteroides* was independently associated with MCI. Graphical modelling also showed a close association between *Bacteroides* and MCI. In conclusion, an increased prevalence of *Bacteroides* is independently associated with the presence of MCI in patients without dementia.

## Introduction

Mild cognitive impairment (MCI) refers to a very early stage of cognitive decline in patients not yet exhibiting dementia and is an important predictive risk factor for dementia^1^. Many people present to health services with MCI, which occurs in up to 20% of people older than 65 years of age^1^, and 10–15% of patients with MCI develop dementia annually^2^. Because the number of patients with dementia has been increasing in Japan, a comprehensive strategy for dementia research has been introduced, which includes analyses of the characteristics of patients with MCI^3^. A multifactorial assessment of MCI can be useful for preventing the progression from MCI to dementia.

Recently, the relationship between the psychophysiological status (such as depression) of a person and their gut microbiome has become an intriguing research focus^4^. In addition, several researchers have identified novel associations between the gut microbiome and dementia^5–7^, suggesting that the gut microbiome may modulate host brain function via a microbiome–gut–brain axis^8^. However, whether the gut microbiome affects cognitive functions has not yet been clarified. A previous study demonstrated the presence of bacterial lipopolysaccharide (LPS) (a component of the outer leaflet of the outer membrane of bacteria) in brain lysates derived from the hippocampus and superior temporal lobe of the neocortex of brains from Alzheimer’s disease (AD) patients^9^. More specifically, Zhao *et al*., reported that bacterial LPS, which is a pro-inflammatory neurotoxin, may be able to cross physiological barriers to access the hippocampus, leading to cognitive impairments^9^, such as memory disturbances. Other studies suggested that the disruption of the neuro-inflammatory system^10^, vascular inflammation^11^, or remote relationships driven by various metabolites^12^ may be involved in the potential mechanisms for cognitive declines caused by the gut microbiome. Specifically, enterobacterial infections may exacerbate AD progression by promoting immune haemocyte recruitment to the brain^10^. Tang *et al*., reported that the presence of bacterial products in the systemic circulation may heighten the inflammatory state^11^. Furthermore, they also reported that microbiota metabolites, such as short-chain fatty acids, trimethylamine/trimethylamine N-oxide, and bile acids, may contribute to life-threatening diseases^11^. Therefore, analysing the gut microbiome in MCI patients without dementia may reveal previously unidentified risk factors for MCI.

We are conducting a clinical study that was designed to investigate the association between the composition of the gut microbiome and a patient’s clinical condition, as assessed using activities of daily living (ADL) scales and cognitive function measures. This study has been named the Gerontological Investigation of Microbiome: a longitudinal estimation study (Gimlet study)^5^ and is being performed at the National Center for Geriatrics and Gerontology (NCGG) (Aichi, Japan). During our baseline analysis, we found that the dysregulation of the gut microbiome, as assessed by terminal restriction fragment length polymorphism (T-RFLP) analysis, which is one of the most well-established and reliable 16S ribosomal RNA-based methods, is cross-sectionally and strongly associated with dementia, independent of traditional dementia biomarkers^5^. Moreover, we found that the presence of dementia, in addition to the presence of cardiovascular risk factors, indicates the advanced dysregulation of the gut microbiome^13^. In the present sub-study of the Gimlet study, we examined the relationship between the gut microbiome and MCI among patients without dementia who had enrolled in the Gimlet study. We hypothesized that there would be differences in the compositions of the gut microbiome between patients with MCI and those with normal cognitive function (NC).

## Results

### Patient characteristics

We previously analysed 128 patients in the Gimlet study. Of these, 46 were excluded: 34 had dementia, and 12 provided an insufficient quantity of faecal samples. Therefore, we analysed the remaining 82 eligible patients without dementia for this sub-analysis (female: 52.4%; mean age: 73.9 ± 8.1 years; mean Mini-Mental State Examination [MMSE] score: 27). Patients were stratified according to their levels of cognitive function and their enterotypes: 61 (74.4%) were classified as MCI, and 21 were classified as NC (Tables 1–3); 38 were enterotype I (46.3%), 5 were enterotype II (6.1%), and 39 were enterotype III (47.6%).

**Table 1:** Demographics of the patients.

	Total			
		MCI	NC	*P*
	(*n* = 82)	(*n* = 61)	(*n* = 21)	
***Demographics***				
Age, years*	76, 68–80	77, 73–81	69, 61–76	<0.001
Female sex, n (%)	43 (52.4)	33 (54.1)	10 (47.6)	0.623
Education, years	12, 9–13	12, 9–12	12, 11–14	0.076
Body mass index, kg/m^2^	22.6, 20.6–24.1	22.5, 20.6–24.5	22.9, 20.5–24.0	0.911
***Risk factors***				
Hypertension, n (%)*	47 (57.3)	39 (63.9)	8 (38.1)	0.045
Diabetes mellitus, n (%)	10 (12.2)	7 (11.5)	3 (14.3)	0.711
Dyslipidaemia, n (%)	36 (43.9)	27 (44.3)	9 (42.9)	1.000
CKD, n (%)	24 (29.3)	20 (32.8)	4 (19.1)	0.278
IHD, n (%)	7 (8.5)	5 (8.20)	2 (9.52)	1.000
History of stroke, n (%)	6 (7.3)	6 (9.84)	0	0.330
Smoking habits, n (%)	24 (29.3)	19 (31.2)	5 (23.8)	0.590
Alcohol consumption, n (%)	33 (40.2)	21 (34.4)	12 (57.1)	0.077
ApoE ε4 carrier, n (%)	18 (22.0)	16 (26.2)	2 (9.5)	0.136

Data are represented as the mean ± standard deviation, median (interquartile range), or number of patients (%).

Wilcoxon signed-rank and χ^2^ tests were used.

The asterisk indicates statistical significance.

Abbreviations: MCI, mild cognitive impairment; NC, normal cognition; CKD, chronic kidney disease; IHD, ischaemic heart disease; ApoE, apolipoprotein E.

**Table 2:** Clinical findings of the patients.

	Total			
		MCI	NC	*P*
	(*n* = 82)	(*n* = 61)	(*n* = 21)	
***Comprehensive geriatric assessment***				
Barthel index	100, 100–100	100, 100–100	100, 100–100	0.159
IADL impairment, n (%)*	29 (35.4)	27 (44.3)	2 (9.5)	0.004
DBDS*	7.5, 3.8–14	10, 4–14.5	2, 0–9	<0.001
GDS*	2.5, 1–5	2, 1–4	4, 2–6	0.032
Vitality index	10, 9–10	10, 9–10	10, 9.5–10	0.118
ZBI*	8.5, 3–18.3	11, 3–20.5	6, 1–9.5	0.043
MNA-SF	13, 11–13	13, 11–13	12, 11–13	0.679
***Cognitive function***				
MMSE*	26.5, 23–29	25, 23–28	29, 27.5–30	<0.001
CDR-SB*	1.0, 0.5–2.5	2, 1–3	0, 0–0.5	<0.001
ADAS-cog*	7.6, 5.3–11.7	8, 5.4–13.3	5.7, 3.25–8	0.003
RCPM*	29, 24–32.5	28, 24–31.5	32, 30–33.75	0.003
FAB*	12, 10–14	11, 9–13	14, 12.5–15.5	<0.001
LM-WMSR I*	10, 5.3–17.5	8, 4–15	16, 8.5–24.5	0.001
LM-WMSR II*	3.5, 0–10	3, 0–8	10, 3–17.5	0.001
***Brain MRI findings***				
SLI, n (%)	3 (3.7)	3 (4.9)	0	0.566
WMH, n (%)*	22 (26.8)	21 (34.4)	1 (4.8)	0.009
CMBs, n (%)	14 (17.1)	12 (19.7)	2 (9.5)	0.502
CSS, n (%)	4 (4.9)	4 (6.6)	0	0.568
VSRAD*	0.85, 0.56–1.42	0.96, 0.65–1.6	0.52, 0.42–0.88	0.010
***Blood flow reduction in SPECT images***				
Posterior cingulate gyrus and/or precuneus, n (%)	53 (67.9)	42 (72.4)	11 (55.0)	0.173
***Arterial stiffness***				
Pulse wave velocity, m/s	17.7, 15.8–21.9	18.2, 16.0–22.3	16.6, 14.8–21.6	0.385
Ankle brachial index	1.10, 1.07–1.15	1.10, 1.04–1.15	1.13, 1.07–1.17	0.406
***Laboratory findings***				
CRP, mg/dL	0.05, 0.02–0.12	0.05, 0.02–0.13	0.03, 0.02–0.10	0.551
eGFR, mL/min/1.73 m^2^*	70.5, 57.5–78.4	63.1, 55.7–74.4	73.5, 68.1–90.1	0.019
***Medication***				
Anti-dementia drug, n (%)	6 (7.3)	6 (9.8)	0	0.330
Anti-hyperglycaemic drug, n (%)	6 (7.4)	4 (6.7)	2 (9.5)	0.647
Anti-hypertensive drug, n (%)*	43 (53.8)	36 (61.0)	7 (33.3)	0.041
Statin, n (%)	28 (35.0)	20 (33.9)	8 (38.1)	0.793
Anti-thrombotic drug, n (%)	15 (18.8)	13 (22.0)	2 (9.5)	0.331
PPI/H2 blocker, n (%)	19 (23.5)	16 (26.7)	3 (14.3)	0.372
Aperient, n (%)	9 (11.1)	7 (11.7)	2 (9.5)	1.000

Wilcoxon signed-rank and χ^2^ tests were used.

Asterisks indicate statistical significance.

Abbreviations: MMSE, Mini-Mental State Examination; CDR-GB, Clinical Dementia Rating Global Score; CDR-SB, Clinical Dementia Rating-Sum of Boxes; ADAS-cog, Alzheimer’s Disease Assessment Scale-Cognitive Subscale; RCPM, Raven’s Coloured Progressive Matrices; FAB, Frontal Assessment Battery; LM-WMSR, Logical Memory subtests I and II of the Wechsler Memory Scale-Revised; IADL, instrumental activities of daily living; DBDS, Dementia Behaviour Disturbance Scale; GDS, Geriatric Depression Scale; ZBI, Zarit Caregiver Burden Interview; MNA-SF, Mini-Nutritional Assessment-Short Form; SLI, silent lacunar infarct; WMH, white matter hyperintensity; CMB, cerebral microbleeds; CSS, cortical superficial siderosis; VSRAD, voxel-based specific regional analysis system for Alzheimer’s disease; SPECT, single photon emission computed tomography; CRP, C-reactive protein; eGFR, estimated glomerular filtration rate; PPI, proton pump inhibitor. Anti-dementia drugs: donepezil, rivastigmine, galantamine, and memantine. Anti-hypertensive drugs: calcium channel blockers, angiotensin-converting-enzyme inhibitors, and angiotensin II receptor blockers.

**Table 3:** Gut microbiome of the patients.

	Total			
		MCI	NC	*P*
	(*n* = 82)	(*n* = 61)	(*n* = 21)	
***Gut microbiota***				
Enterotype*				0.009
Enterotype I	38 (46.3)	34 (55.7)	4 (19.1)	
Enterotype II	5 (6.1)	4 (6.6)	1 (4.8)	
Enterotype III	39 (47.6)	23 (37.7)	16 (76.2)	
F/B ratio	1.33, 0.72–2.17	1.22, 0.65–2.21	1.65, 1.10–2.14	0.096

Wilcoxon signed-rank and χ^2^ tests were used.

The asterisk indicates statistical significance.

Abbreviations: F/B ratio, Firmicutes/Bacteroidetes ratio. Enterotype I: Bacteroides >30%; Enterotype II: Prevotella >15%; enterotype III: others.

### MCI vs. NC

Compared with NC patients, those with MCI were older (MCI *vs*. NC: median years, 77 *vs*. 69, *P* < 0.001), had a higher prevalence of hypertension (63.9% *vs*. 38.1%, *P* = 0.045) and were more likely to present with indications of cerebral small vessel disease (SVD), such as white matter hypersensitivity (WMH) (34.4% *vs*. 4.8%, *P* = 0.009), and impaired instrumental ADL scores (44.3% *vs*. 9.5%, *P* = 0.004). Additionally, patients with MCI had higher scores on the Dementia Behaviour Disturbance Scale (DBDS), Zarit Caregiver Burden Interview (ZBI), and voxel-based specific regional analysis system for Alzheimer’s Disease (VSRAD) (median scores: 10 *vs*. 2, *P* < 0.001; 11 *vs*. 6, *P* = 0.043; and 0.96 *vs*. 0.52, *P* = 0.010, respectively), as well as lower Geriatric Depression Scale (GDS) scores (median score: 2 *vs*. 4, *P* = 0.032) and reduced cognitive function, as demonstrated by lower scores on the MMSE, Raven’s Coloured Progressive Matrices (RCPM), Frontal Assessment Battery (FAB), and Logical Memory subtests I and II of the Wechsler Memory Scale-Revised (LM-WMSR) (median scores: 25 *vs*. 29, *P* < 0.001; 28 *vs*. 32, *P* = 0.003; 11 *vs*. 14, *P* < 0.001; 8 *vs*. 16, *P* = 0.001; and 3 *vs*. 10, *P* = 0.001, respectively) and higher scores on the Clinical Dementia Rating Scale-Sum of Boxes (CDR-SB) and Alzheimer’s Disease Assessment Scale-Cognitive Subscale (ADAS-cog) (median scores: 2 *vs*. 0, *P* < 0.001; and 8 *vs*. 5.7, *P* = 0.003, respectively). Patients with MCI had more enterotype I microbes and fewer enterotype III microbes than patients with NC (55.7% *vs*. 19.1%; and 37.7% *vs*. 76.2%, respectively, *P* = 0.009), suggesting a higher prevalence of *Bacteroides* and a lower prevalence of ‘other’ bacteria (Tables 1–3).

### Comparison by enterotype

As described, we stratified the enrolled patients according to the enterotypes of their microbes and performed the following comparisons: enterotype I *vs*. non-enterotype I and and enterotype III *vs*. non-enterotype III (Tables S1 and S2, respectively). When compared with non-enterotype I patients, patients with increased levels of enterotype I microbes were more likely to exhibit a higher prevalence of cerebral SVD components (enterotype I *vs*. non-enterotype I: silent lacunar infarcts [SLI], 5.3% *vs*. 2.3%, *P* = 0.594; WMH, 39.5% *vs*. 15.9%, *P* = 0.024; cerebral microbleeds [CMB], 23.7% *vs*. 11.4%, *P* = 0.155; and cortical superficial siderosis [CSS], 7.9% *vs*. 2.3%, *P* = 0.332), higher VSRAD scores (median score, 0.96 *vs*. 0.80, *P* = 0.112), lower global cognitive function (median score, MMSE, 25 *vs*. 27, *P* = 0.046; CDR-SB, 2 *vs*. 0.5, *P* = 0.002), and impaired memory function (LM-WMSR I, 8 *vs*. 11, *P* = 0.134; and LM-WMSR II, 2 *vs*. 5, *P* = 0.041). No significant differences were observed for demographics or risk factors (e.g., mean age, 77 *vs*. 75, *P* = 0.145) between enterotype I patients and non-enterotype I patients (Table S1). In contrast, compared with non-enterotype III patients, patients with more enterotype III microbes had a lower prevalence of cerebral SVD components (enterotype III *vs*. non-enterotype III, SLI, 2.6% *vs*. 4.7%, *P* = 1.000; WMH, 15.4% *vs*. 37.2%, *P* = 0.045; CMB, 10.3% *vs*. 23.3%, *P* = 0.148; and CSS, 2.6% *vs*. 7.0%, *P* = 0.617) but were more likely to have low VSRAD scores (median score, 0.69 *vs*. 1.00, *P* = 0.048) and increased global cognitive function (median score, MMSE, 27 *vs*. 25, *P* = 0.071; and CDR-SB, 0.5 *vs*. 2, *P* = 0.007) (Table S2). Although there appeared to be significant differences regarding the prevalence of hypertension and alcohol consumption between enterotype III and non-enterotype III patients, no significant differences were observed for the *Firmicutes*/*Bacteroidetes* (F/B) ratio (Tables S3 and S4). No significant differences were observed for enterotypes between males and females (Table S5).

### Multivariable analysis

Multivariable logistic regression analyses were performed to identify factors that are independently associated with MCI. Due to the small number of patients, these analyses were performed in step-by-step increments of the number of independent variables: model 1 (adjusted for age, sex, education year, apolipoprotein E [ApoE] ε4 carrier, enterotype, and F/B ratio), model 2 (stepwise adjusted for model 1 and prevalence of risk factors), and model 3 (stepwise adjusted for model 2, magnetic resonance imaging [MRI] findings, and single photon emission computed tomography [SPECT] findings). Multivariable logistic regression analyses revealed that increased levels of enterotype I microbes was associated with the presence of MCI, independent of age, sex, education years, ApoE ε4, traditional risk factors, and brain imaging (model 1: odds ratio [OR 10.2], 95% confidence interval [95% CI], 2.23–62.7, *P* = 0.002; model 2: OR 5.95, 95% CI, 1.61–28.2, *P* = 0.006; and model 3: OR 5.36, 95% CI, 1.30–28.7, *P* = 0.019; Table 4). Conversely, enterotype III was independently associated with the presence of MCI (model 1: OR 0.09, 95% CI, 0.02–0.42, *P* = 0.002; model 2: OR 0.19, 95% CI, 0.05–0.65, *P* = 0.008; and model 3: OR 0.19, 95% CI, 0.05–0.65, *P* = 0.008) (Table 5).

**Table 4:** Multivariable logistic regression analysis for the presence of MCI (adjusted by enterotype I).

	OR	95% CI	*P*
***Model 1***			
Age, years*	3.89	1.76–10.3	<0.001
Female sex	0. 67	0.17–2.40	0.541
Education, years	0.86	0.39–1.89	0.706
ApoE ε4 carrier*	11.9	1.91–132.8	0.006
Enterotype I*	10.2	2.23–62.7	0.002
F/B ratio	1.26	0.90–2.00	0.195
***Model 2***			
Age, years*	3.86	1.87–9.38	<0.001
ApoE ε4 carrier*	9.41	1.58–98.9	0.011
Enterotype I*	5.95	1.61–28.2	0.006
***Model 3***			
Age, years*	4.66	1.95–14.3	<0.001
ApoE ε4 carrier*	10.8	1.68–129.7	0.010
Smoking habits	3.10	0.80–14.3	0.104
Enterotype I*	5.36	1.30–28.7	0.019
WMH	7.33	0.84–174.2	0.075
CMBs	0.16	0.01–1.72	0.124

Abbreviations: CI, confidence interval; OR, odds ratio; ApoE, apolipoprotein E; F/B ratio, Firmicutes/Bacteroidetes ratio; WMH, white matter hyperintensity; CMB, cerebral microbleeds.

The asterisk indicates statistical significance.

Model 1: adjusted for age, sex, education years, ApoE ε4 carrier, enterotype, and F/B ratio.

Model 2: stepwise adjusted for model 1 and prevalence of risk factors (hypertension, diabetes mellitus, dyslipidaemia, chronic kidney disease, ischaemic heart disease, history of stroke, smoking habit, and alcohol drinking habit).

Model 3: stepwise adjusted for model 2, magnetic resonance imaging findings (presence of silent lacunar infarcts, white matter hypersensitivity, cerebral microbleeds, cortical superficial siderosis, and voxel-based specific regional analysis system for Alzheimer’s disease scores), and single photon emission-computed tomography findings (presence or absence of a reduction in blood flow in the area of the posterior cingulate gyrus and/or precuneus).

**Table 5:** Multivariable logistic regression analysis for the presence of MCI (adjusted by enterotype III).

	OR	95% CI	*P*
***Model 1***			
Age, years*	3.46	1.58–8.97	0.001
Female sex	0. 82	0.22–2.95	0.766
Education, years	0.83	0.37–1.85	0.643
ApoE ε4 carrier*	13.4	2.08–156.3	0.004
Enterotype III*	0.09	0.02–0.42	0.002
F/B ratio	1.37	0.94–2.34	0.115
***Model 2***			
Age, years*	3.68	1.80–8.81	<0.001
ApoE ε4 carrier*	9.65	1.63–100.3	0.01
Enterotype III*	0.19	0.05–0.65	0.008
***Model 3***			
Age, years*	3.68	1.80–8.81	<0.001
ApoE ε4 carrier*	9.65	1.63–100.3	0.010
Enterotype III*	0.19	0.05–0.65	0.008

Abbreviations: CI, confidence interval; OR, odds ratio; ApoE, apolipoprotein E; F/B ratio, Firmicutes/Bacteroidetes ratio; WMH, white matter hyperintensity; CMB, cerebral microbleeds.

The asterisk indicates statistical significance.

Models 1–3 were adjusted using the same formula in Table 4.

### Graphical modelling

**Figure 1:**
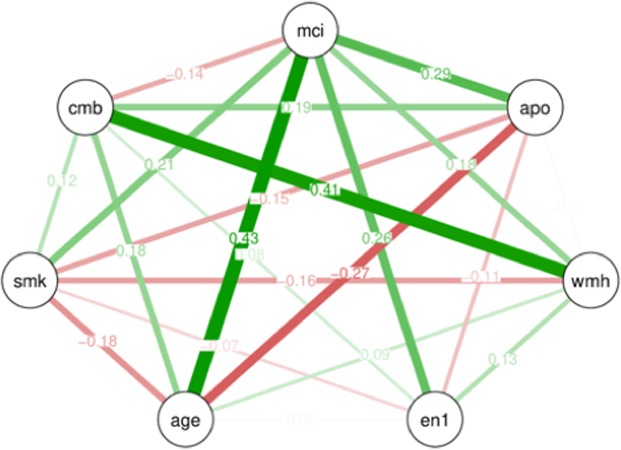
Graphical modelling of factors used for the multivariable analyses. Line thickness is proportional to the number of patients that contributed to the comparison. Green lines indicate a positive relationship, and red lines indicate a negative relationship. Abbreviations: mci, mild cognitive impairment; apo, apolipoprotein E ε4 carrier; wmh, white matter hyperintensity; en1, enterotype I; age, patient’s age; smk, smoking habit; cmb, cerebral microbleeds.

Graphical modelling was performed to visualize the mutual associations among factors used in the multivariable logistic regression analyses. Accordingly, modelling showed that the presence of MCI was more likely to be associated with age and was equally associated with ApoE ε4 and enterotype I. Enterotype I was closely associated with the presence of MCI when compared with the other factors. Furthermore, a close association between CMB and WMH was shown by this modelling (Fig. 1).

## Discussion

The primary finding of our present study was that the increased prevalence of *Bacteroides*, defined as enterotype I, was independently associated with the presence of MCI in patients without dementia. Specifically, patients with an increased prevalence of *Bacteroides* were more likely to have lower global cognitive function (indicated by MMSE and CDR scores) and impaired memory dysfunction (indicated by logical memory subtests) compared with non-enterotype I patients, although there were no significant differences in demographics and risk factors, such as age, sex, and hypertension.

Recent studies have reported controversial findings regarding an association between the gut microbiome and dementia. For example, previous reports have shown both decreased^5,6^ and increased^7^ proportions of *Bacteroides* in patients with dementia. Vogt *et al*.^7^, speculated that LPS is the component of *Bacteroidetes* that potentiates systemic inflammation and amyloid fibrillogenesis, ultimately resulting in amyloid deposition. Furthermore, Zhao and Lukiw *et al*., also reported that microbiome-derived LPS was enriched in the perinuclear region of the AD brain^14^, whereas LPS was detected in the hippocampus of patients with AD^9^. Our findings are consistent with these studies because our patients with more *Bacteroidetes* had lower logical memory subtest scores, indicating impaired memory function.

The mechanism through which the gut microbiome affects human cognitive functions remains unknown, although animal studies strongly implicate the gut microbiome as a key regulator of the brain and behaviour^15^. The functional pathways through which the gut microbiome communicates with the brain, also known as gut–microbiome–brain cross-talk, is a bidirectional, functional communication network between microbes and the brain that comprises neuroendocrine, neural, and neuroimmune signalling pathways^16^. Explicitly, three hypothesis have been proposed: (1) a circulation pathway, indicating the transportation of microbial metabolites, toxins, and pro-inflammatory factors; (2) a neuroendocrine pathway, indicating the activation of neuroendocrine cells that can be attributed to microbial metabolites; and (3) a neural pathway, indicating an interaction between the gut microbiome and the autonomic nervous system. This previous report also showed that an increase in the abundance of a pro-inflammatory gut microbiome taxon and a reduction in the abundance of an anti-inflammatory taxon may be associated with a peripheral inflammatory state in patients with cognitive impairments and brain amyloidosis^17^. Another report describing the gut–brain module analysis of faecal metagenomes identified the microbial synthesis of metabolites and suggested a potential role for these metabolites in the onset of depression^18^.

At present, we do not have sufficient data to identify the underlying mechanism that mediates the association between the gut microbiome and cognitive impairment. However, potential differences regarding the presence of WMH may represent supporting evidence that cerebral SVD acts as an intermediate between cognitive impairment and the gut microbiome. Likewise, Ong *et al*., reported that alterations in brain structure, as assessed by diffusion tensor imaging of MRI scans, were associated with the gut microbiome^19^, suggesting there may be a definite and direct relationship between the gut microbiome and the brain parenchyma. Further studies examining the association between cerebral SVD and gut microbiome metabolites in the Gimlet study cohort will shed light on this issue.

Here, we found other interesting findings. There was an independent association between WMH and the gut microbiome, although other components of cerebral SVD, such as SLI and CMB, did not show such significant differences. This finding suggests that a potential mechanism regarding the development of WMH may be attributable to the gut microbiome, either directly or indirectly. Further study regarding the association between cerebral SVD and the gut microbiome is warranted.

Our study has several strengths. First, we systematically assessed the cognitive functions of patients, using a comprehensive geriatric assessment in the setting of the memory clinic. These steps have provided detailed analyses of cognitive function. Second, we found a close and strong association between the gut microbiome and MCI, identified by multivariable analyses and graphical modelling, compared with the relationships between the other risk factors and MCI. Applying graphical modelling has strengthened our findings. Third, our comprehensive assessment regarding the gut microbiome and MCI may fill the knowledge gap regarding the mechanisms that connect cognitive decline with the gut microbiome. Finally, because we have established a new relationship and have widened the knowledge of such associations with regard to MCI, our findings may contribute to a better understanding and increased attention being paid to the associations between the gut microbiome and cognitive functions.

Nonetheless, this study has several limitations. A causal relationship between the gut microbiome and MCI could not be established because of the cross-sectional study design. The small number of patients renders our study at risk of being statistically underpowered. The unequal numbers of patients with MCI and those with NC may also be a potential limitation. Selection bias may exist because this was a single hospital-based cohort. Furthermore, potentially confounding factors may exist because the gut microbiome is affected by diet^20,21^ and the presence of complications, such as blood pressure variability and cardiovascular disease^13^. In particular, a recent study^21^ suggests that the modified Mediterranean-ketogenic diet can modulate the gut microbiome and metabolites in association with improved biomarkers regarding Alzheimer’s disease. Likewise, we plan to assess the nutritional status including the diet pattern in the future study because the Japanese diet pattern can alter the intestinal bacteria and have beneficial effects on humans^20^.

Assessments of the amyloid-β precursor protein may be useful because this factor suggests inflammatory endothelial dysfunction and the risk of cognitive impairment^22^. The specific mechanism through which the microbiome affects cognitive decline has not yet been clarified. Rather than identifying the roles of specific bacteria, the functional analysis of the gut microbiome as one integrated organ may be more practically useful. Our data indicate both accelerating (in the present study) and decelerating (in a previously published study^5^) effects of *Bacteroides* on cognitive decline. However, this relationship may be attributable to unidentified taxa (indicated by ‘other’) and/or metabolites, which may depend on the intraintestinal environment. Next-generation sequencing technologies could be useful for identifying the specific genera or species of microbes that are collectively categorized as ‘other’ bacteria by the T-RFLP method.

To date, various risk factors for cognitive impairment have been proposed, such as aging, education years, hypertension, diabetes mellitus, and social factors^1^; however, the human gut microbiome has not previously been mentioned as a risk factor. Our findings add the gut microbiome as a new risk factor for cognitive impairment. More importantly, controlling the gut microbiome may represent a possible method for the prevention and intervention of cognitive impairment.

Although this sub-analysis study contains a small number of patients and our analysis is preliminary, our findings provide supporting evidence for a relationship between the gut microbiome and cognitive impairment. Longitudinal assessments of the gut microbiome using next-generation sequencing technology, assessments of the various metabolites produced by the gut microbiome, and assessments of diet patterns should be investigated in future studies to clarify the underlying mechanism that connects the gut microbiome with cognitive function.

## Conclusions

We showed that components of the gut microbiome, in particular *Bacteroides*, may be associated with the presence of MCI in patients without dementia. We speculate that some gut microbiome metabolites could affect cognitive functions through a microbiome–gut–brain axis. Further studies are warranted to examine such relationships.

## Methods

### Study design

This study was a sub-analysis of our previously published, single-centre observational study (Gimlet study)^5^. The aim of this study was to investigate the association between the gut microbiome and MCI in patients without dementia. This study complied with the Declaration of Helsinki and was approved by the Institutional Review Board at the National Center for Geriatrics and Gerontology (No. 1191). Informed consent was obtained from all patients and their families before participation in this study. The Gimlet study is registered with the UMIN Clinical Trials Registry (UMIN000031851). Detailed information has been provided in the supplementary file and in the previous report^5^.

### Subjects

Patients were eligible for the Gimlet study if they met the following criteria: (1) were able to undergo a brain MRI; (2) provided informed consent in writing; (3) provided informed consent for the NCGG Biobank to store their clinical data, blood, and faecal samples; and (4) were accompanied by a study partner who could assess the patient’s condition. Patients were excluded if they met the following criteria: (1) were unable to undergo an MRI examination or the MRI scan could not be evaluated because of movement; (2) had local lesions, such as cerebral infarctions, that were detected by MRI before enrolment and that could significantly affect cognitive function; (3) had a history of a major psychological disorders or current, serious or unstable alcohol or drug abuse; (4) had ≤6 years of education; (5) had a history of cancer of the digestive tract; or (6) were judged by an investigator to be ineligible to participate as a study subject (e.g., recent use of antibiotics, brain tumour, encephalitis/meningitis, normal pressure hydrocephalus, Huntington’s disease). Patients were also excluded from this sub-analysis if they met the following criteria: (1) had dementia; or (2) were unable to provide sufficient faecal samples to facilitate the analysis of metabolites both now and in the future.

### Baseline assessment

All participants underwent a comprehensive geriatric assessment^23^ using the following: (1) demographic characteristics; (2) risk factors, such as hypertension, dyslipidaemia, diabetes mellitus, ischaemic heart disease, chronic kidney disease, smoking habits, or a history of stroke and alcohol consumption; (3) basic and instrumental ADL scales, assessed using the Barthel Index^24^ and Lawton and Brody scale^25^; (4) global cognitive function, assessed using the MMSE^26^ and CDR^27^; (5) neuropsychological testing, using the ADAS-cog^28^, RCPM^29^, FAB^30^, and LM-WMSR^31^; (6) laboratory variables, including ApoE ε4 as a risk factor for AD; (7) ankle brachial index and pulse wave velocity, as indicators of arteriosclerosis^32^ and the ‘impact’ of pulse^33^; (8) brain imaging, such as MRI and SPECT; (9) an assessment of other factors, such as the presence of frailty^34^ and subjective hearing loss; and (10) an assessment of social and lifestyle factors, e.g., using the Mini-Nutritional Assessment-Short Form (MNA-SF) to assess nutritional status^35^. Clinical samples and data were provided by the NCGG Biobank, which collects clinical data for research.

### Classification of cognitive function

In the Gimlet study^5^, patients were divided into two categories, using measures that reliably indicate the presence of dementia: (1) a group without dementia (MMSE ≥ 20 and CDR < 1), and (2) a group with dementia (MMSE < 20 and/or CDR ≥ 1). In the present sub-study, the patients without dementia were further divided into two categories: MCI and NC. MCI was defined as MMSE ≥ 20 and CDR = 0.5, indicating possible, very mild dementia and suggesting that the patient has a higher risk of developing dementia^36^. NC was defined as MMSE ≥ 20 and CDR = 0.

### Brain imaging

Patients underwent a 1.5 T MRI of the brain (Philips Ingenia, Eindhoven, the Netherlands). MRI scans were obtained, including diffusion-weighted imaging, fluid-attenuated inversion recovery imaging, T2-weighted imaging, T2^*^-weighted gradient echo imaging, 3D T1-weighted sagittal and axial coronal views, and 3D time-of-flight MR angiography scans. The presence and components of cerebral SVD were categorised using standards for reporting neuroimaging vascular changes^37^, including SLI, WMH, CMB, and CSS. Moreover, VSRAD advance software (Eisai Co., Ltd., Tokyo, Japan) was used to quantify cortical and hippocampal atrophy, using standardised z-scores. Increased VSRAD scores suggest the potential presence of AD, as this score reflects hippocampal atrophy, one of the characteristics of the AD brain^38^. Patients also underwent N-isopropyl-p-[^123^I]-iodoamphetamine-SPECT. The presence or absence of reduced blood flow in the area of the posterior cingulate gyrus and/or the precuneus was assessed as a surrogate marker of AD^39^.

### Gut microbiome

Faecal samples were collected at home, just after evacuation, by patients or their family members, using scoop collection tubes, while patients were consuming their usual diets, and samples were frozen and preserved at −81 °C at the NCGG Biobank. After all samples were collected, the gut microbiome was analysed by the TechnoSuruga Laboratory (Shizuoka, Japan), using T-RFLP analysis^40^. T-RFLP analysis is one of the most well-established and reliable 16S ribosomal RNA-based methods, especially when considering its high throughput and reproducibility. First, T-RFLP was used to classify gut microbes into the following 10 groups: *Prevotella*, *Bacteroides*, Lactobacillales, *Bifidobacterium*, *Clostridium* cluster IV, *Clostridium* subcluster XIVa, *Clostridium* cluster IX, *Clostridium* cluster XI, *Clostridium* cluster XVIII, and ‘others’. Second, by referencing the Human Faecal Microbiome T-RFLP profile^41,42^, the gut microbiome was stratified into three enterotypes: enterotype I included *Bacteroides* at >30%, enterotype II included *Prevotella* at >15%, and enterotype III included the remaining bacteria. Third, the F/B ratio was examined, as an increase in the F/B ratio is indicative of dysbiosis^43^. The phylum Firmicutes included the Lactobacillales and *Clostridium* clusters, and the phylum Bacteroidetes included *Bacteroides* and *Prevotella*.

### Statistical analysis

Continuous, ordinal, and categorical variables are expressed as the mean ± standard deviation, median and interquartile range, and frequency or proportion (percentage), respectively, and were compared using an unpaired Student’s *t*-test, a Wilcoxon rank-sum test, and a χ^2^ test, respectively. First, patients were divided into two groups, according to the presence or absence of MCI, and their clinical characteristics and the compositions of their gut microbiomes were compared using the Wilcoxon rank-sum test and χ^2^ test, respectively. Second, patients were also divided into two groups, either enterotype I and non-enterotype I, or enterotype III or non-enterotype III, to compare their clinical characteristics based on Gimlet baseline data. This division indicated that there were a small number of patients with enterotype II. Third, multivariable logistic regression models were performed to identify factors that were independently associated with MCI. Backward stepwise multivariable logistic regression analyses were performed by adjusting for patient demographics, ApoE ε4, gut microbiome (enterotype and the F/B ratio), risk factors, brain MRI findings, and blood flow reductions on SPECT images. Last, graphical modelling was generated to illustrate mutual associations among the factors used in the multivariable logistic regression analyses, including traditional risk factors and gut microbiome, to visualize and compare mutual relationships. Appropriately, ORs are presented as 95% CIs. All comparisons were two-tailed, and P < 0.05 was considered to represent statistical significance. Data were analysed using the JMP 11.0 software package (SAS Institute Inc., Cary, NC, USA), and graphical modelling was generated using R (R Language and Environment for Statistical Computing, Vienna, Austria).

## Supplementary information

Supplemental_file

## Data Availability

The datasets used and/or analysed during the current study are available from the corresponding author on reasonable request.
